# Rubber Dam Isolation for Bonding Ceramic Veneers: A Five-Year Post-Insertion Clinical Report

**DOI:** 10.7759/cureus.20748

**Published:** 2021-12-27

**Authors:** Carlos A Jurado, Nicholas G Fischer, Mohammed E Sayed, Jose Villalobos-Tinoco, Akimasa Tsujimoto

**Affiliations:** 1 Prosthodontics, Woody L. Hunt School of Dental Medicine, Texas Tech University Health Sciences Center, El Paso, USA; 2 Minnesota Dental Research Center for Biomaterials and Biomechanics, School of Dentistry, University of Minnesota, Minneapolis, USA; 3 Prosthetic Dental Sciences, College of Dentistry, Jazan University, Jazan, SAU; 4 Oral Rehabilitation, School of Dentistry, Autonomous University of Queretaro, Querétaro, MEX; 5 Operative Dentistry, College of Dentistry, University of Iowa, Iowa City, USA

**Keywords:** dentistry, isolation, rubber dam, bonding, esthetics, ceramic veneers

## Abstract

It has been well-documented that uncontaminated ground enamel provides the most predictable substrate for the bonding of ceramic veneers, and thus conservative tooth preparation with complete isolation using a rubber dam is key to the long-term success of the restorations presented with five years of follow-up. Rubber dam isolation provides several advantages to the clinician, such as preventing contamination of the working field by saliva, blood, and sulcular fluids, and improving direct visibility. However, it may be a challenge to the younger clinician to properly isolate teeth prior to bonding ceramic veneer. The present case report demonstrated the sequence and some clinical tips for a case in which the rubber dam is placed from a second premolar to the opposite second premolar and held with clamps, the rubber dam is gently invaginated into the sulcus, and clamps are selected and placed on each tooth to create an ideal situation for the adhesion of the ceramic veneer. This step-by-step sequence may help the younger clinician in understanding how to gently manage soft tissue in order to properly provide complete isolation with rubber dam for future bonding of ceramic veneers. Following these methods, the clinician can achieve complete isolation, invaginate the rubber dam in the sulcus without causing tissues to bleed, and reduce the time needed for bonding procedures.

## Introduction

A rubber dam is a device for the isolation of the working field and is commonly used for restorative and endodontic procedures [1]. Its use has been criticized for being time-consuming [2] and costly, however, the placement of a rubber dam is fundamental for antisepsis and moisture control as well as protecting patients from inhalation of toxic materials [3]. Dr. Sanford Christie Barnum was the first clinician to use a thin piece of rubber in order to isolate teeth in 1869 [4]. The quality of tooth isolation is important to prevent leakage in both directions, the operation field and the oral cavity [5]. The popularity of rubber dam use was described as widespread just three years later [6,7]. However, rubber dam usage decreased at the beginning of the 20th century partly because of the development of suction devices and silver amalgam. As a result, the use of cotton rolls and alcohol were recommended as isolation alternatives [7]. Despite the revived efforts to remind dentists of the importance of rubber dam, most clinicians today are mainly concerned with efficiency and profitability in the clinic [8].

Over the years, many researchers have investigated the reasons for the non-usage of the rubber dam and improvements that might lead to its acceptance [9,10]. The results show that clinicians do not use the rubber dam because they believe it is challenging, time-consuming, cumbersome, and that patients will not accept it. Currently, it seems that those arguments against the use of rubber dams have not changed even though these views are not supported by recent literature [11,12].

Placing a rubber dam prior to bonding ceramic restorations aims to prevent any contamination and maximize the bonding properties between the ceramic and the tooth surface. Unfortunately, there are no reports on how to achieve full isolation efficiently and without damaging soft tissues. This case report aimed to provide clinical tips and examples on how to properly place a rubber dam prior to bonding of ceramic veneer restorations in the esthetic zone. Following these methods, the clinician can achieve complete isolation, invaginate the rubber dam in the sulcus without causing tissues to bleed, and reduce the time needed for bonding procedures.

## Case presentation

Rubber dam isolation is a meticulous process and the clinician needs to be aware of how to gently handle the tissue during its placement in order to achieve an ideal working field. Improper management of clamps on soft tissue will cause the gingiva to bleed, creating a non-ideal situation for bonding protocols.

A 34-year-old female Hispanic patient presented with a chief complaint of "My teeth are short and I want to improve my smile.” In the review of her medical history, she was medically fit and visited her physician on regular basis. She presented a dental history of few posterior teeth restorations that were done five years ago. Her oral hygiene was good, well-maintained, and classified as philosophical per the House classification.

**Figure 1:**
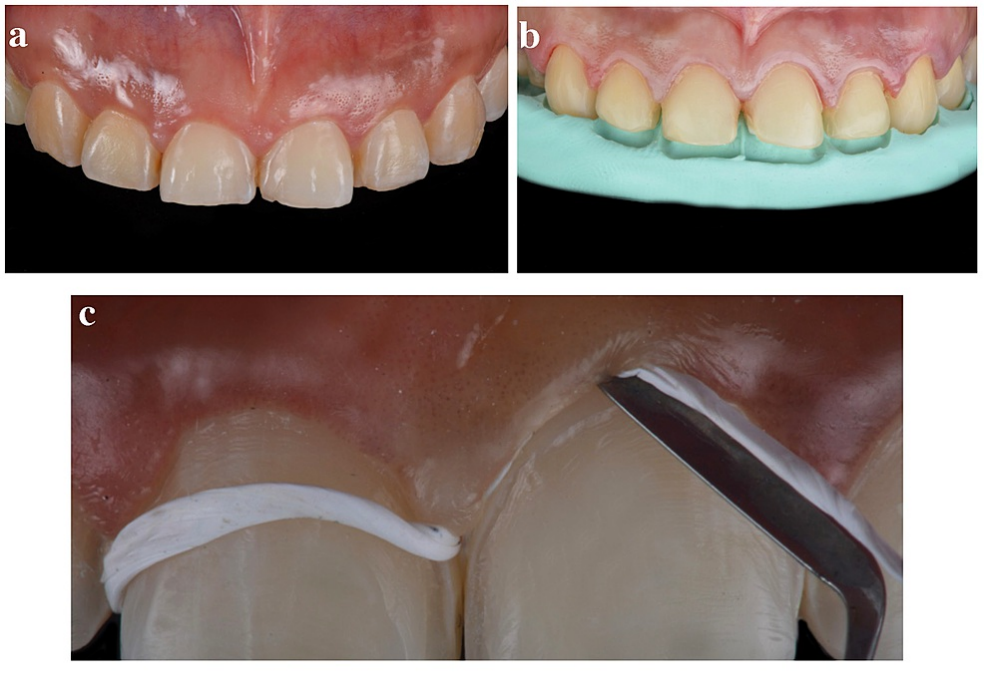
Initial preparation and Teflon tape The image is showing (A) initial intra-oral view, (B) final conservative tooth preparations, and (C) packing of Teflon tape.

After initial evaluation and conservative tooth preparations (Figures 1a, 1b), Teflon tapes (Loctite thread seal tape; USA: Henkel Loctite Corp.) were gently packed using the cord packer instrument (113 serrated gingival cord packer; Chicago, IL: Hu-Friedy) around the neck of all teeth that were to receive a bonded ceramic restoration (Figure 1c). Teflon tape was used, compared to conventional retraction cords, because of Teflon's flexibility. The rubber dam (dental dam; Bucharest, Romania: Nic Tone) was perforated to at least two teeth posterior of the teeth ready to receive the final bonded restoration.

**Figure 2:**
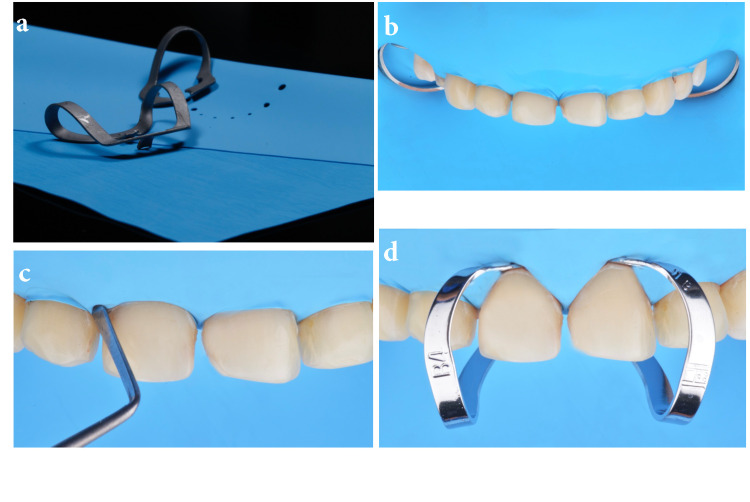
Rubber dam preparation and clamps The image is showing (A) rubber dam and clamps selection, (B) holder clamps on premolars, (C) invaginating rubber dam with cord packer and air pressure, and (D) clamps on teeth to prior bonding ceramics.

The rubber dam needed to be placed from the second premolar to the second premolar of the same arch as ceramic veneers from canine to canine were to be bonded (Figure 2a). Holder clamps (rubber dam clamps #2; Chicago, IL: Hu-Friedy) were placed on the second premolars in order to maintain the rubber dam in the ideal position (Figure 2b). The rubber dam was invaginated with a hand instrument cord-packer (113 serrated gingival cord packer) and gentle application of air. The instrument needed to follow the neck of the tooth allowing the air pressure to invaginate the rubber dam (Figure 2c). In addition, clamps (hygenic brinker clamp B4; Cuyahoga Falls, OH: Coltene/Whaledent Inc.) were placed on the specific teeth to which restorations were eventually bonded (Figure 2d).

**Figure 3:**
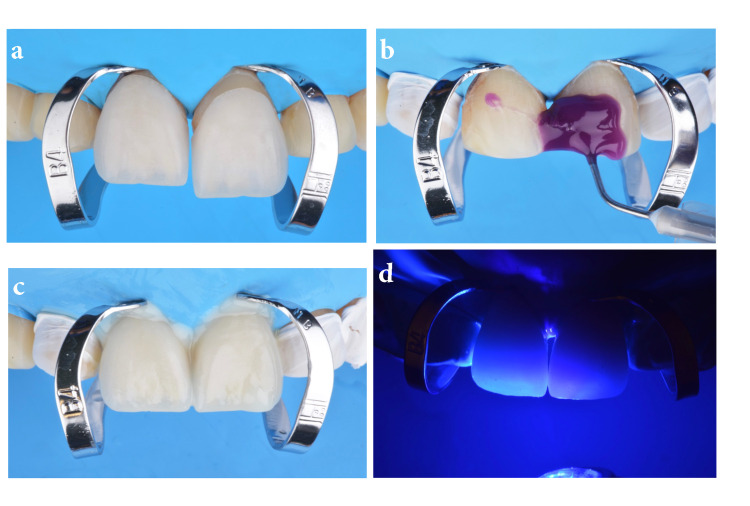
Try-in, etching, and restoration placement The image is showing (A) try-in prior bonding protocol, (B) etching treatment, (C) placing final restorations, and (D) light curing.

At this stage, a dry try-in of the ceramic restoration was important in order to evaluate the clear tooth margin access prior to bonding the restorations (Figure 3a). Ideally, the restorations are cemented in pairs starting from the midline to the distal, so clamps on #8 and #9 were placed, and then the tooth surface was treated with 32% phosphoric acid gel (Uni-Etch w/BAC; Schaumburg, IL: Bisco Dental) for 30 seconds and then rinsed and gently dried (Figure 3b). Then primer and adhesive were applied (OptiBond FL; Orange, CA: Kerr Dental) following the manufacturer’s instructions and light-cured (Valo LED curing light; South Jordan, UT: Ultradent Products Inc.) for 20 seconds. A light color cement (Variolink Esthetic LC; Schaan, Liechtenstein: Ivoclar Vivadent) was applied to both veneers for #8 and 9, which were placed onto the teeth (Figure 3c). Excess cement was removed with a brush (Profi natural bristle brush; Hilzingen, Germany: Renfert) and floss (Oral-B Glide pro-health deep clean floss; Cincinnati, OH: Procter & Gamble) over the interproximal surfaces before light curing for 20 seconds on the facial, 20 seconds on mesial, 20 seconds on distal, and 20 seconds on the incisal surface (Figure 3d).

**Figure 4:**
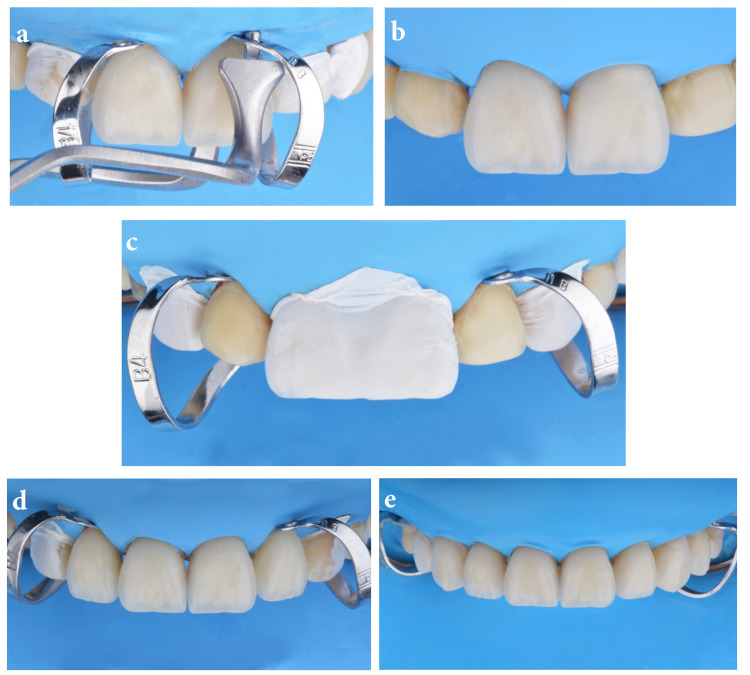
Clamp removal and lateral incisors The image is showing (A) clamps removal after bonding central incisor veneers, (B) clamps removed from central incisors, (C) isolation of centrals and canines, (D) clamps placement on lateral incisors, and (E) final bonded restorations.

Clamps were removed with clamp forceps (four rubber dam clamp forceps; Chicago, IL: Hu-Friedy) (Figures 4a, 4b). Lateral incisors and canines were isolated with Teflon tape prior to clamp placement (hygenic brinker clamp B4) on both lateral incisors (Figure 4c) and the same bonding protocol sequence was followed for the teeth and veneers on #7 and #10, then #6 and #11, and finally #5 and #12 (Figures 4d, 4e).

**Figure 5:**
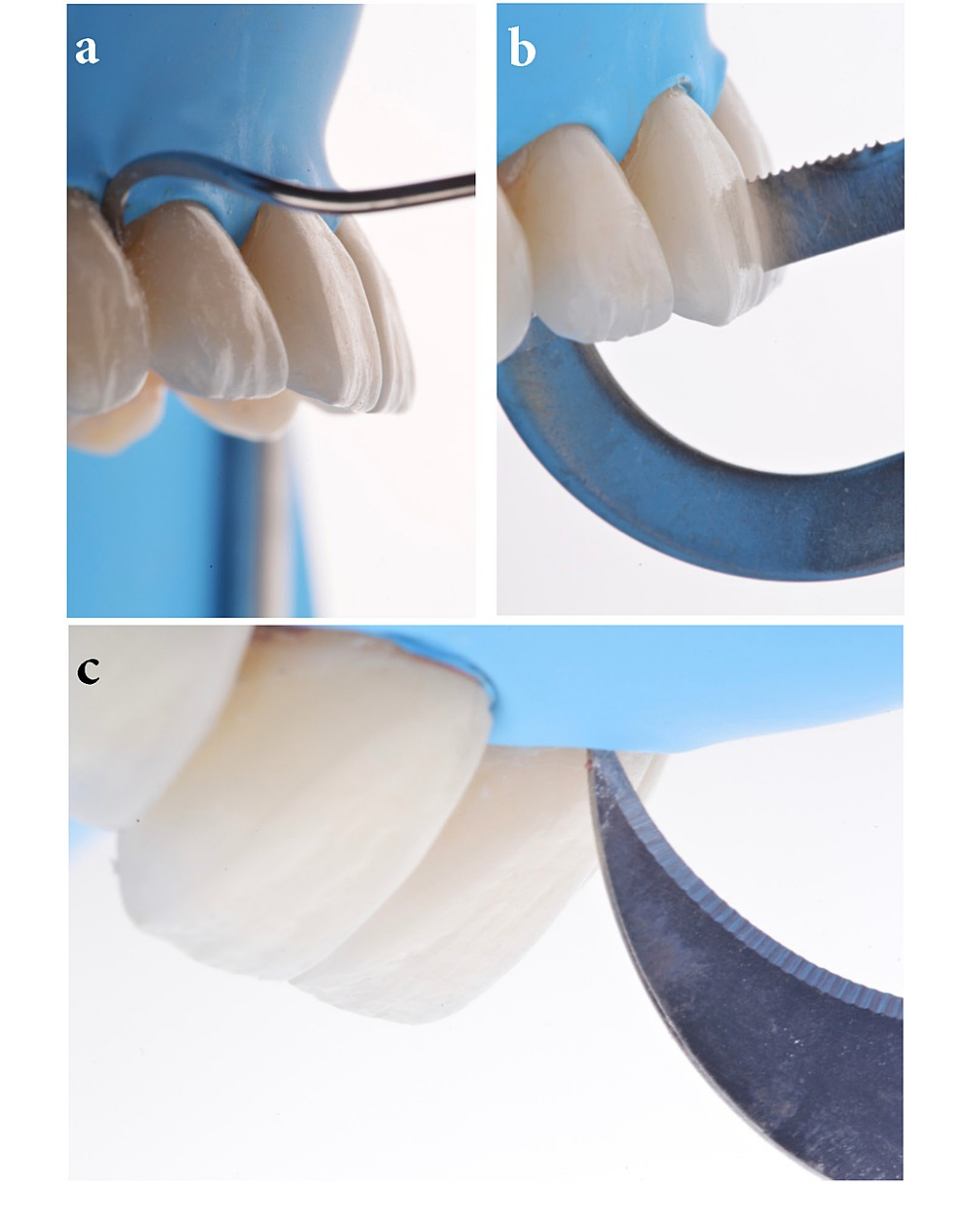
Excess removal of cement The image is showing (A) removing excess cement on cervical surfaces, (B) excess removal of cement on interproximal surfaces, and (C) excess removal of cement with a blade.

Excess cement was gently removed from the cervical and interproximal areas with a hand instrument scaler (LM-Arte Eccesso; Helsinki, Finland: Planmeca), interproximal matrix, and a #12 blade (surgical scalpel blade no. 12; Charlotte, NC: Salvin Dental Specialties) (Figures 5a-5c).

**Figure 6:**
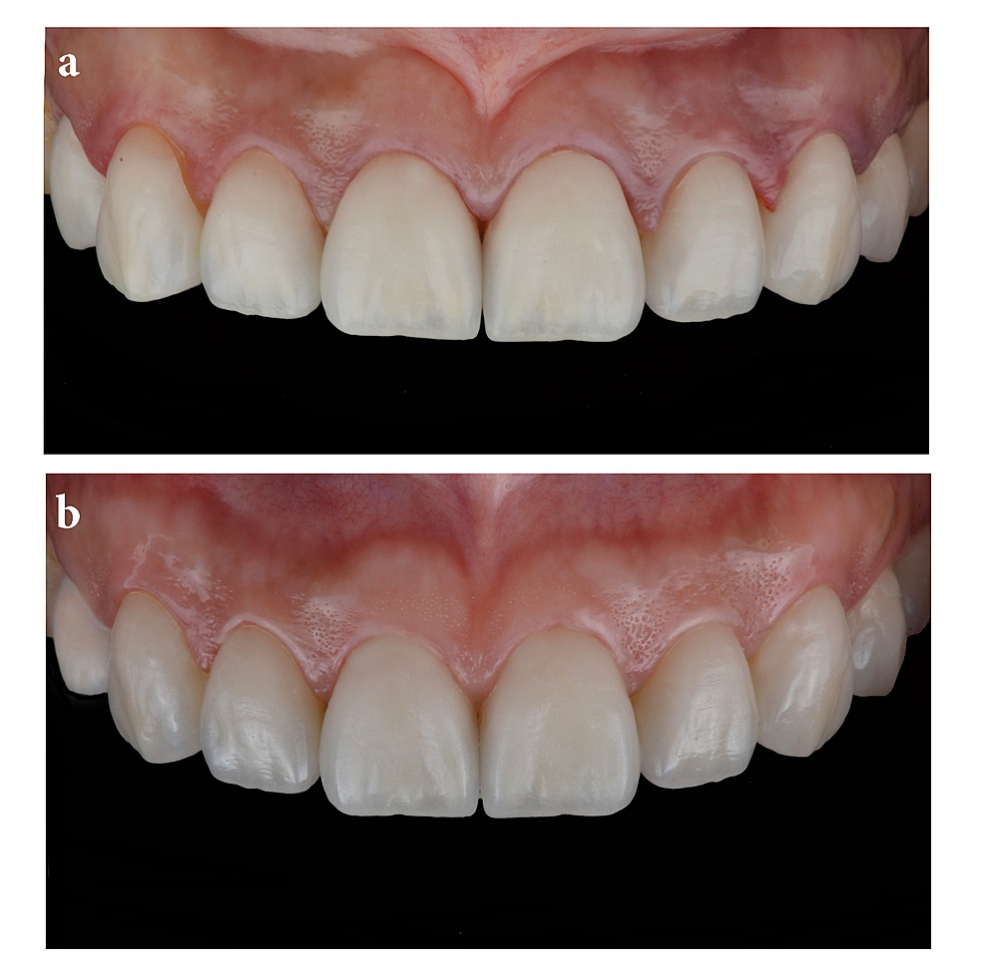
Final restoration and five-year follow-up The image is showing (A) final restorations following the bonding procedure and (B) intra-oral view of final restorations five years following cementation.

Glycerin gel was then applied to the ceramic surfaces to prevent the development of an oxygen inhibition layer (liquid strip; Schaan, Liechtenstein: Ivoclar Vivadent) and the surfaces were again light-cured for 20 seconds each. The final outcome fulfilled patient esthetic demands (Figure 6a). An occlusal guard was provided to wear at night to prevent any damage to the restorations. A follow-up at five years again demonstrated a successful overall case (Figure 6b).

## Discussion

The use of rubber dams has been a controversial issue; however, it is necessary to use one to achieve the best possible results of bonding protocols. A Cochrane Library review from 2016 found that, at least for direct restorations, rubber dams lead to a lower failure rate [8]. However, further research, with better design, was suggested in this review. Indeed, the authors are unable to find a published study directly comparing long-term outcomes with regard to rubber dams usage and bonded ceramic veneers. Future research is necessary. Overall, rubber damns have been reported to protect the airway, reduce aerosol contamination, ensure an aseptic working area, and of most interest to us, protect bonding areas from saliva [1].

Unfortunately, rubber dams are not used often by dental practitioners in many countries, even though they are recommended by many dental associations [13,14]. Clinicians not using the rubber dam present the argument that it is a time-consuming protocol, but studies have shown that the average time for rubber dam placement is between one and two minutes [2]. As a result, the use of a rubber dam does not significantly prolong the treatment time, nor does the removal of a rubber dam, which usually takes 10-15 seconds, cause a significant prolongation of the treatment time [15,16]. Another disadvantage claimed by clinicians that dislike the use of rubber dams is patient discomfort, but there are many studies showing that patients have reported a higher level of comfort during dental treatment with a rubber dam than during treatment without a rubber dam [11,12]. None of the factors observed in those studies, such as age, gender, or previous experience with dental treatment with a rubber dam showed a statistically significant influence on the reported comfort. 

Clinicians need to be aware of the type of material the rubber dam is made of because patients can have allergic reactions to some materials, most notably latex. The common symptoms of latex allergy include skin reactions such as itching, redness, rash, or hives, itchy nose, throat, or eyes, nausea, abdominal cramps, and difficulty breathing. While most reactions occur within minutes of exposure, some skin reactions may not develop for 24-48 hours afterward [17]. Anaphylaxis, which could be a life-threatening condition, can occur and can cause a drop in blood pressure. The clinician needs to ask patients about latex allergy history, and in case of doubt, the patient should be referred to a physician for testing.

A common problem may be leakage around the neck of the teeth after placing the rubber dam. This could be due to many factors such as incorrect selection of sizes for the holes in the dam, incorrect space selection between the punched holes, non-ideal selection of the clamps, or lack of good invagination of the rubber dam. It is important to punch the appropriate hole size for the teeth and to space the punch holes at least 1/4" apart. If the hole size is too large, there will be leakage at the necks of the teeth, and if there is not enough space between holes, then there will not be enough interseptal dam material to cover the interdental papilla and to invert the dam on either side. The latter problem can also occur if the holes are too small, leading to the dam being too tight around the tooth and tearing.

Placing a piece of floss around the neck of the tooth and then crossing the floss on the facial surface and gently tucking the floss gingivally into the sulcus provides a good invagination of the rubber dam and it is recommended to invert the rubber dam in the whole of the working area. However, the clinician must be aware that the patient may need to be anesthetized in order to prevent any soft tissue sensitivity caused by the flossing. Here, we used Teflon tape instead of gingival retraction cord because Teflon tape is more flexible than retraction cord and comes in variable thicknesses. Generally, Teflon tape is useful to the dentist's repertoire for a variety of techniques, such as landmark work by others [18].

Many clinicians are unaware that there are clamps specifically designed to be gingival retractors, such as those used for class V restorations or, as in the example provided here, for bonding ceramic veneers. Their dull and blunted design allows them to be placed into the sulcus without damaging the soft tissue. Clinicians may certainly try to use other types of clamps in order to isolate teeth, but their design may not let them retract the soft tissue and they may damage the gingiva.

Placing the rubber dam prior to bonding ceramic veneers may present many challenges such as gingival irritation and/or bleeding. Clinicians need to gently invaginate the rubber dam and place the ideal clamps on teeth to support the rubber dam and for bonding the ceramic restoration. The discussed points are not hard to apply and lead to an effective and efficient placement of the rubber dam. To summarize, the ideal size of holes in the rubber dam is chosen; the rubber dam is placed from the second premolar to the opposite second premolar and held with clamps; the rubber dam is gently invaginated in the sulcus; ideal individual clamps are selected and placed on each tooth for the adhesion of the ceramic veneer. Obviously, clinicians can bond final restorations without having total isolation with a rubber dam, but even minimal contamination may compromise the effectiveness of the bonding agents. Moreover, clinicians working without a rubber dam may need meticulous help from several dental assistants.

## Conclusions

The use of conservative protocols, such as minimal tooth preparations in enamel and complete isolation with rubber dam prior to bonding ceramic restorations, maximize the bonding system properties, such as those shown here for ceramic veneers. The goal of this case report was to spotlight how proper complete isolation with a rubber dam prevented contamination of the working field by saliva, blood, and sulcular fluids around the neck of the tooth. Ideal hole size and space between holes - like that used here - were important in order to put the rubber dam in an ideal position. This case demonstrated how gentle invagination of the rubber dam with air, floss, and Teflon tape was crucial to prevent bleeding of the soft tissues. Finally, proper gingival retractor clamp selection was indispensable in order to separate the rubber dam and subgingival margin of the tooth. These results culminated in patient satisfaction five years post-insertion.
